# The Role of Circulating Biomarkers in Peripheral Arterial Disease

**DOI:** 10.3390/ijms22073601

**Published:** 2021-03-30

**Authors:** Goren Saenz-Pipaon, Esther Martinez-Aguilar, Josune Orbe, Arantxa González Miqueo, Leopoldo Fernandez-Alonso, Jose Antonio Paramo, Carmen Roncal

**Affiliations:** 1 Laboratory of Atherothrombosis, Program of Cardiovascular Diseases, Cima Universidad de Navarra, 31008 Pamplona, Spain; gsaenzdepip@alumni.unav.es (G.S.-P.); josuneor@unav.es (J.O.); japaramo@unav.es (J.A.P.); 2 IdiSNA, Instituto de Investigación Sanitaria de Navarra, 31008 Pamplona, Spain; esthermartinezaguilar@hotmail.com (E.M.-A.); amiqueo@unav.es (A.G.M.); leopoldofa@gmail.com (L.F.-A.); 3 Departamento de Angiología y Cirugía Vascular, Complejo Hospitalario de Navarra, 31008 Pamplona, Spain; 4 CIBERCV, Instituto de Salud Carlos III, 28029 Madrid, Spain; 5 Laboratory of Heart Failure, Program of Cardiovascular Diseases, Cima Universidad de Navarra, 31008 Pamplona, Spain; 6 Hematology Service, Clínica Universidad de Navarra, 31008 Pamplona, Spain

**Keywords:** peripheral arterial disease, biomarkers, inflammation, coagulation, extracellular vesicles, microRNAs, machine learning

## Abstract

Peripheral arterial disease (PAD) of the lower extremities is a chronic illness predominantly of atherosclerotic aetiology, associated to traditional cardiovascular (CV) risk factors. It is one of the most prevalent CV conditions worldwide in subjects >65 years, estimated to increase greatly with the aging of the population, becoming a severe socioeconomic problem in the future. The narrowing and thrombotic occlusion of the lower limb arteries impairs the walking function as the disease progresses, increasing the risk of CV events (myocardial infarction and stroke), amputation and death. Despite its poor prognosis, PAD patients are scarcely identified until the disease is advanced, highlighting the need for reliable biomarkers for PAD patient stratification, that might also contribute to define more personalized medical treatments. In this review, we will discuss the usefulness of inflammatory molecules, matrix metalloproteinases (MMPs), and cardiac damage markers, as well as novel components of the liquid biopsy, extracellular vesicles (EVs), and non-coding RNAs for lower limb PAD identification, stratification, and outcome assessment. We will also explore the potential of machine learning methods to build prediction models to refine PAD assessment. In this line, the usefulness of multimarker approaches to evaluate this complex multifactorial disease will be also discussed.

## 1. Introduction

The term peripheral arterial disease (PAD) includes a range of non-coronary arterial syndromes that are caused by an alteration in the structure and function of the arteries supplying the brain, visceral organs, and extremities. Numerous pathophysiological processes can contribute to the formation of stenosis or aneurysms in the non-coronary circulation, but atherosclerosis is the most common lesion that affects the aorta and its branches [1,2]. In this review, we will focus on lower extremity PAD referring to the chronic lower limb ischemia of atherosclerotic origin.

It has been estimated that PAD affects 12–14% of the general population, approximately 202 million people across the world [2,3]. Its prevalence increases with age, affecting around 10–25% of people older than 55 years, and 40% of those older than 80 years, being associated with significant morbidity, mortality, and quality of life impairment [4,5].

PAD, frequently accompanied by atherosclerosis in other vascular beds, exhibits higher risk of ischemic events and death compared to other cardiovascular (CV) pathologies. Likewise, coronary artery disease (CAD) is present in approximately 60–80% of patients with PAD, whereas 12–25% suffer accompanying carotid artery stenosis [3,5]. In the REACH (Reduction of Atherothrombosis for Continued Health) study 4.7% of PAD patients suffered from concomitant coronary disease, 1.2% from concurrent cerebrovascular disease, and 1.6% presented both. Similarly, about one-third of men and one- quarter of women with known coronary or cerebrovascular disease are diagnosed with PAD [6]. Moreover, the severity of PAD is also associated to the prevalence of CAD. Conversely, left main coronary artery stenosis and multivessel CAD are independent predictors of PAD, and patients with PAD exhibit more advanced coronary atherosclerosis [2]. As a consequence, PAD patients present a 20–60% higher risk of myocardial infarction, and a 2–6 fold higher risk of death due to a coronary event [5,7], while the risk of stroke increases by approximately 40% [3,5]. The ARIC (Atherosclerosis Risk in Communities) study conducted among men with PAD showed 4–5 times higher risk of having a stroke or a transient ischemic attack than those without PAD, although in women, the association was not significant [8]. Indeed, it has been recently described that PAD is an equivalent risk factor to CAD for CV death [5].

Individual risk estimation of PAD is based on the Fontaine or Rutherford classification in combination with the ankle-brachial pressure index (ABI), which is the current gold standard for vascular severity categorization. The aforementioned scales divide lower extremity PAD into two major groups: namely patients with intermittent claudication (IC, Fontaine I and II), the most common clinical presentation at early stages associated to mild symptoms, and chronic limb-threatening ischemia (CLI, Fontaine III and IV) including patients at more advanced stages that can develop ischemic ulcerations or gangrene of the foot [9]. The ABI is a non-invasive test able to detect arterial occlusive disease in symptomatic patients, but more importantly, also in asymptomatic patients, presenting a sensitivity of 95% and a specificity of 100% compared with arteriography when assessing PAD vs controls. ABI values ≤0.90 are generally considered diagnostic of PAD although so far, no strong evidence supports its relationship with CV morbi-mortality or a reduction in thrombotic events and death by the treatment of screening-detected PAD patients [4]. Despite the high discriminative power of ABI for stenotic plaques, it is not useful to identify patients at the initial phases of the disease, when atherosclerotic lesions are not big enough to promote a hemodynamically significant stenosis. Moreover, in the subgroup of diabetic patients ABI loses sensitivity due to arterial calcification, often rendering values above 1.4, that have not been correlated clinically with increased CV morbidity and mortality [4]. In consequence, PAD is often undiagnosed and untreated, especially in the early stages of the pathology or in diabetic patients, presenting silent rates of about 50% [5], highlighting the need of early diagnosis and prognosis markers, and novel therapeutic targets for lower limb PAD.

**Figure 1:**
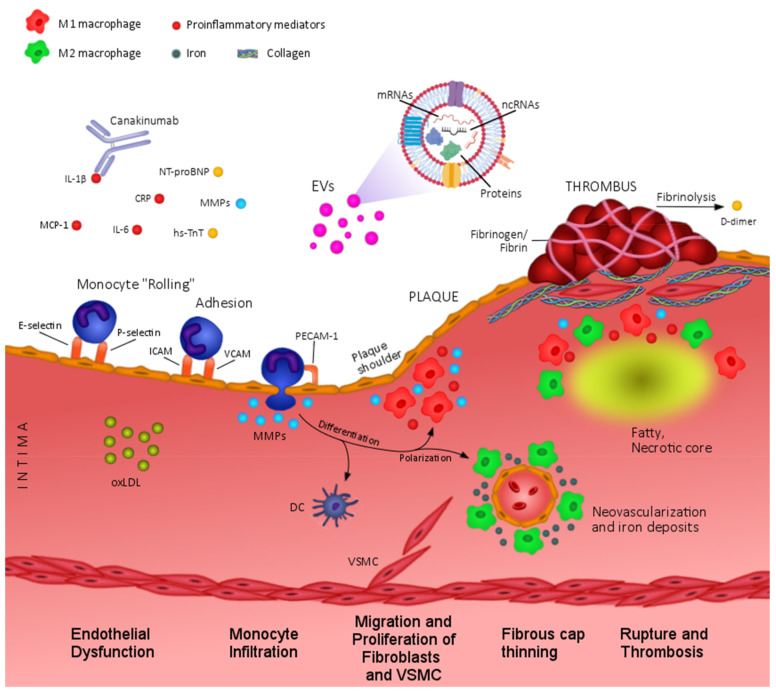
Circulating biomarkers in atherosclerosis and PAD pathophysiology. At early stages of atherosclerosis, endothelial dysfunction leads to increased levels of proinflammatory cytokines (IL1β, IL6, CRP, MCP-1), extracellular vesicles (EVs), proteases (MMPs), and adhesion molecules (E-selectin, P-selectin, ICAM, VCAM,) that contribute to monocyte recruitment and infiltration into the intima. Microenvironmental factors such as MMPs orchestrate plaque progression by regulating macrophage polarization towards proinflammatory (M1) or anti-inflammatory (M2) phenotypes, which are predominantly located in unstable areas (i.e.,: plaque shoulder) or neovascularization regions, respectively. In advanced lesions, inflammatory factors and MMPs exacerbate fibrous cap thinning and contribute to plaque instability, rupture, and formation of fibrin clot. Extracellular vesicles (EVs), mainly of platelet origin, also participate in thrombus formation according to the exposure of procoagulant factors (e.g.,: tissue factor and phosphatidylserine). In addition, reduced tissue perfusion due to arterial stenosis and thrombosis might cause increased levels of tissue damage-related biomarkers such as NT-proBNP or hs-TnT. CRP, C reactive protein; DC, dendritic cells; ICAM, intercellular adhesion molecule; IL, interleukin; MCP-1, monocyte chemoattractant protein; MMPs, matrix metalloproteinases; NT-proBNP, N-terminal pro-brain natriuretic peptide; oxLDL, oxidized Low Density Lipoproteins; hs-TnT, high sensitivity troponin T; VCAM, vascular cell adhesion molecule; VSMC, vascular Smooth Muscle Cells.

Considering the lack of precise biomarkers for PAD assessment, and the key role of inflammation and vessel remodelling in atherosclerotic plaque progression and rupture, several authors have evaluated the levels of proteins related to those pathological processes in this regard (Figure 1). The circulating concentrations of some cytokines (C reactive protein (CRP) or Interleukin (IL)-6), coagulation factors (D-dimer or fibrinogen), proteases (Matrix metalloproteinases (MMPs) and their inhibitors, tissue inhibitors of metalloproteinases (TIMPs)) or cardiac damage markers have been reported to be increased in PAD patients according to pathological stages and CV complications [10,11,12,13], while other oxidative stress markers, and angiogenesis-related molecules rendered inconclusive results [12]. Most interestingly, extracellular vesicles (EVs) and non-coding RNAs are emerging as novel biomarkers and/or biological effectors in PAD pathophysiology [14,15]. Whether these candidates will improve the risk estimation associated to traditional CV risk factors is still unclear and will need to be further evaluated, not only by single biomarker assessment, but also and more interestingly, by combining groups of biomarkers and clinical parameters. Machine learning (ML) offers the possibility to model large datasets integrating all biological variables [16]. By doing so, these algorithms build prediction models aiming to help clinicians to better diagnose and estimate CV risk also in the context of PAD.

In this review, we will summarize the current status of different sets of circulating biomarkers, EVs, and non-coding RNAs for lower extremity PAD diagnosis and outcome assessment. Moreover, we will also discuss how the implementation of multimarker approaches and machine learning methods can produce more accurate disease classification and prediction models.

## 2. Inflammation and Coagulation Biomarkers in PAD

Low grade inflammation has been involved in all the phases of PAD, from atherosclerosis initiation to progression, and from plaque rupture to thrombosis. Accordingly, in the last decades several inflammatory and haemostatic molecules have been evaluated as possible biomarkers for PAD assessment, although it still remains controversial how or whether they will be able to outperform traditional CV risk factors [17,18] (Table 1). CRP, an acute phase reactant, is one of the most studied inflammatory molecules for PAD evaluation. Early in 2001, Ridker PM et al. reported the use of CRP as a potential marker of incident PAD [19], which was later confirmed by other authors [14,20]. In addition, several prospective studies have reported increased levels of CRP in PAD patients compared to controls [14,21,22,23,24] and an association with PAD severity and ABI [25,26,27]. CRP has also been proposed as a marker of worse outcome considering major CV events (stroke and myocardial infarction), major amputation/revascularization and mortality in high risk PAD patients [14,23,28], although it has been suggested that CRP might be more useful for short-term risk prediction rather than for long-term evaluation [29,30]. In this line, a meta-analysis by Singh TP et al. including studies with samples sizes ranging from 51 to 1157 patients reported an associated hazard ratio of 2.26 (1.65–3.09) for the categorized CRP variable and CV events and death in a follow-up ≤2 years [11]. Similarly, Kremers B et al. comprising 13 studies found that patients with increased CRP levels had a relative risk of 1.86 (1.48–2.33) for major adverse cardiovascular events (MACE), and of 3.49 (2.35–5.19) for mortality [10]. These evidences suggest the potential use of CRP for PAD diagnosis and prognosis. It is worth considering however, that some of the summarized papers were conducted with a limited number of patients (Table 1), and that in many cases risk prediction was estimated in the short term, rather than in the long term.

The impact of other inflammatory biomarkers for PAD diagnosis and prognosis has been also evaluated (Table 1). For instance, IL-6, IL-8, pentraxin-3, neutrophil gelatinase-associated lipocalin (NGAL), calprotectin or tumor necrosis factor (TNF)-α were significantly higher in PAD patients compared with healthy controls [14,21,22,23,24,33,34], and some of these candidates; IL-6, TNF-α, and pentraxin-3 were associated to PAD severity, assessed either by ABI or clinical scales [21,23,52]. Among those pro-inflammatory markers, IL-6 stands out as a prominent predictor of functional outcomes. In the Edinburgh Artery Study, IL-6 showed more consistent and stronger independent predictive value than CRP and soluble adhesion molecules for PAD progression [27]. As such, initial levels of IL-6 showed an association with ABI changes at five and 12 years of follow-up, while CRP was only associated with ABI changes at 12 years [27]. Similarly, Murabito JM et al. described that among different inflammatory molecules, namely CD40L, CRP, monocyte chemoattractant protein (MCP)-1, and myeloperoxidase, only IL-6 and TNF receptor (TNFR)-2 remained significantly associated with hemodynamic or clinical PAD after adjustment for confounding factors [26]. Levels of inflammatory biomarkers have been also explored in relation to lower limb functional impairment in PAD patients with claudication, showing an inverse correlation between high levels of IL-6 and TNF-α with the maximal walking time [22]. In line with these results, increased blood concentrations of CRP and IL-6 were significantly correlated with poorer six-minute walk performance in PAD [35]. Recently Russell KS et al. reported that reducing inflammation with an anti-IL-1β neutralizing antibody (canakinumab) improved walking performance in PAD patients and IC [36]. The canakinumab treated patients presented a reduction in blood CRP and IL-6, that was more significant and consistent for circulating IL-6 compared to CRP during the follow-up [36]. Regarding in-stent restenosis, Ueki Y et al. found no differences in the maximum change of IL-6, MCP-1, and TNF-α between patients with and without restenosis with a mean follow-up of 1 year [53], while a latest study by Guo S et al. reported an independent association between the pre- and post-operative (24 h) IL-6 levels and six-month in-stent restenosis, while for CRP the association was only found with the 24 h postintervention levels [32]. In summary, these data support a prominent role of inflammation in PAD, specially of IL-6, and suggest that its pharmacological modulation, even if indirect, might be a therapeutic alternative for PAD patients. Larger studies should be performed to corroborate the possible use of IL-6 as a biomarker for PAD and/or as pharmacological target.

The lack of reliable biomarkers in PAD has extended the study to adhesion molecules, selectins, and haemostatic candidates rendering dissimilar results. Higher circulating levels of soluble intercellular adhesion molecule (ICAM)-1, vascular cell adhesion molecule (VCAM)-1, E-Selectin, L-Selectin, P-Selectin, neopterin, serum amyloid A, and D-dimer have been reported in PAD patients compared to control groups, while for fibrinogen only a moderate increase was found [22,24,31,33,34,37]. Another study reported differences in blood levels of P-Selectin, platelet factor 4, VCAM, thrombin-antithrombin complex, pro-thrombin fragments 1+2, and D-dimer only when assessing CLI vs control, but not when IC was examined [38], whereas Beckman JA et al. found no differences at all in VCAM-1 and ICAM-1 levels between PAD patients and controls [23]. Similarly, blood concentrations of P-selectin, ICAM-1, and fibrinogen have been significantly related to both ABI and clinical PAD in the Framingham Offspring Study participants [26], while other authors found no such associations [23,37]. In the Edinburgh Artery Study, only ICAM-1, but not VCAM-1 or E-selectin, was independently correlated with changes in ABI at 12 years of follow-up [27]. Additionally, a slower fast-paced walking speed was associated to higher levels of VCAM-1, ICAM-1 and D-dimer in PAD patients [22,35]. Regarding other outcomes, increased fibrinogen levels were associated with subsequent ischemic events and major bleeding at the FRENA (Factores de Riesgo y Enfermedad Arterial) registry [39] and with mortality risk in other studies [40,41]. Similarly, D-dimer, the degradation product of crosslinked fibrin, was increased in PAD patients suffering ischemic heart disease compared to the non-event group [42], and was associated with all-cause mortality within 1 and 2 years of follow-up [30]. Finally, the neutrophil-to-lymphocyte ratio (NLR), obtained from the hemogram data, has been shown to be predictor of PAD diagnosis [43,44,45,54] and poor outcome [46,55]. Ertuk M et al. described a two-fold increase in CV mortality risk in IC and CLI patients presenting NLR>3 [47]. Moreover, in patients undergoing endovascular intervention high preoperative NLR has been independently associated to post-procedural restenosis [48], major adverse limb events (MALE) and death [49,50,51,54]. Despite the abundant evidences gathered in the literature, the role of adhesion and hemostatic molecules for PAD diagnosis and prognosis still remains unclear. One of the main limitations might be related to the disparity in the recruited patient numbers among different studies (Table 1). The NLR however, easier to calculate from the hemogram, seems a promising candidate in PAD assessment, although risk prediction stratification would benefit from a comparable NLR cut-off point in different scenarios.

Multimarker approach: PAD is a multifactorial disease and single biomarker determination might not be able to completely reflect the complex pathophysiological processes underlying vascular remodeling. Moreover, different inflammatory proteins might represent distinct molecular pathways operating through different mechanisms [56]. In consequence, it has been proposed that a multimarker approach might be more useful for PAD evaluation [10]. For instance, in the ARIC study the addition of galectin-3 and hs-CRP to traditional atherosclerotic predictors improved the risk prediction of PAD incidence [20]. Regarding PAD severity, Egnot NS et al. identified two biomarker groups associated to low ABI; one consisting of the inflammatory markers CRP, IL-6 and fibrinogen, and the second including the coagulation markers D-dimer and pentraxin-3 [57]. Surprisingly coagulation markers presented a stronger association with lower ABI compared to inflammatory molecules [57]. As such, the relative risk for cardiovascular mortality on IC, but not on CLI, was five times higher when considering the combination of α-defensin and CRP than when assessing either α-defensin, or CRP alone [25]. In addition, a recent report from our lab shows that the combination of calprotectin and CRP increased the risk for amputation and CV mortality when compared with each protein independently [14]. These reports suggest that single biomarker approaches might be too simplistic to predict complex multifactorial diseases such as PAD, and urge the discovery of those molecular partners that in combination might render the best outcome for PAD assessment.

## 3. MMPs/TIMPs in PAD

MMPs are a family of zinc-dependent enzymes that catalyse the proteolysis of extracellular matrix proteins, being negatively regulated by the TIMPs (−1 to −4) that directly bind to their catalytic domain. MMPs are produced by many inflammatory cells participating in numerous physiological and pathological processes. In atherosclerosis, MMPs dysregulation is associated with leukocyte infiltration, vascular smooth muscle cell (VSMC) migration and plaque formation. Moreover, MMPs seem to be involved in vascular remodelling, intimal thickness, and lumen narrowing during restenosis after endovascular treatment of atherosclerotic lesions.

Circulating MMPs are being increasingly recognized as biomarkers of atherosclerosis and CV risk. In PAD, MMPs have been implicated in the inflammatory process of atherosclerosis, degrading collagen and allowing VSMC migration within the vessel wall, leading to vessel occlusion and ischemia. In a large community-based study, patients with previously undetected ABI ≤ 0.9 presented higher levels of the MMP-2/MMP-9 ratio compared to non-PAD control subjects (1.4 > ABI > 0.9) [58], and high levels of both gelatinases, MMP-2 and -9, were also reported in diagnosed PAD patients compared to controls [24,34]. As for other MMPs: MMP-1, -3, -7, -10, -12, and -13 were elevated in PAD patients vs. controls, while TIMP-1 levels were lower [59,60]. Furthermore, MMP-8, -9, -10, and -13 significantly correlated with lipid levels, and MMP-10 with age and hypertension in PAD patients [59]. These data suggest that MMPs may be associated with PAD development, although to corroborate that the combined measurement of MMPs and ABI will be able to improve the diagnosis and posterior treatment of PAD, further long-term and larger studies should be performed.

Moreover, patients with CLI, the most severe form of PAD, had increased MMP-10 and TIMP-1 levels compared with IC, and those in the highest MMP-10 tertile presented an elevated incidence of mortality, either all-cause or CV [60]. In line with this results, Tayebjee MH et al. observed higher MMP-9 and TIMP-1 levels in CLI patients compared with IC, that correlated with white cell count, whereas no differences were reported in circulating TIMP-2 [61]. It has been proposed that the observed rise in circulating TIMP-1 in CLI could be related to the increased proteolytic activity of vascular patients, or reflect the enhanced fibrosis shown by these subjects [60,61]. Likewise, skeletal muscles of CLI patients presented increased mRNA and protein levels of MMP-9, -19, TIMP-1 and -2 compared to controls, whereas MMP-2 rendered inconclusive results [62]. In experimental models of hind limb ischemia, the levels and activity of MMP-2, -9, and -10 significantly increased in crural muscle after femoral artery ligation [63,64,65], and MMP-9 deficiency resulted in reduced tissue perfusion. The role of MMP-9 in arteriogenesis and angiogenesis still remains controversial. As such, Meisner JK et al. reported decreased necrotic and fibroadipose tissue clearance in MMP-9 knockout mice after femoral artery ligation despite normal arteriogenic and angiogenic vascular growth [66], while other authors described reduced capillary density and impaired EPC mobilization in absence of MMP-9 [67,68]. In addition, MMP-10 deficiency resulted in increased skeletal muscle necrosis and inflammatory cell infiltration early after femoral ischemia, that resulted in delayed muscle recovery at the regenerative phase [65]. These data suggest the contribution of the MMPs/TIMPs system to the pathophysiology of PAD.

Endothelial dysfunction is a key process leading to atherosclerosis and PAD, and the organism tries to counterbalance its progress by activating endothelial progenitor cell (EPC) mobilization and homing to the sites of vessel injury to induce repair. EPCs mobilization from the bone marrow is triggered by inflammation and MMPs activity. As such, Morishita T et al. investigated the pattern of EPCs mobilization and their association with inflammation and oxidative stress markers in patients with PAD [69]. They reported an increase in the number of circulating EPCs in the moderate phases of PAD, that decreased in the advanced phases of the disease, and was negatively correlated with the expression of membrane type-1 MMP (MT1-MMP) on peripheral blood mononuclear cells. MT1-MMP is an important regulator of EPC mobilization and angiogenesis [70] that cleaves CD44 adhesion molecule and reduces bone marrow stromal and progenitor cells interaction from bone marrow. These data suggest that the biphasic response of EPCs in PAD pathogenesis could be associated with changes in MT1-MMP expression [69], although its role as a potential biomarker in PAD needs to be confirmed in larger cohorts.

Vascular complications, including PAD, are more frequent among diabetics, and thus it has been hypothesized that MMPs are preferentially activated in patients with both pathologies. Increased plasma levels and zymographic activity of MMP-2 and MMP-9 has been shown in patients with type II diabetes, regardless their vascular status, in comparison with normal volunteers [71]. However, when comparing diabetic subjects with and without PAD, only plasma MMP-2 zymographic activity was higher in those presenting both pathologies vs. diabetes alone, while for MMP-9 activity no differences were observed [71]. Supporting these results, Chung AWY et al. reported an upregulation of MMP-2 and MMP-9 gene expression and gelatinolytic activity in mammary arteries of diabetic patients, that correlated positively with that of angiostatin, an antiangiogenic molecule, and negatively with VEGF, contributing likewise to impair blood vessel formation and PAD development in diabetic patients [72]. Similarly, a study with a larger sample size of type 2 diabetic patients (n = 302) reported elevated levels of MMP-2 in patients with PAD and diabetes, compared to non-PAD diabetics, which was accompanied with an increase in elastin degradation products (ELM), suggesting the regulation of MMP-2 and ELM by hyperglycemia in patients with PAD [73].

Endovascular surgery (angioplasty/stent) has become the first election therapy for most patients with PAD. However, balloon inflation and stent placement induced arterial wall damage may alter MMPs expression, contributing to constrictive remodelling, intimal thickening and re-stenosis [74,75]. In symptomatic PAD patients undergoing elective lower limb percutaneous revascularization (angioplasty/stent) the periprocedural profile of circulating MMP-2, -3, -7, and -9 and TIMP-1 and -2 has been documented. Compared to admission values, there was a significant elevation in serum MMP-3 and -7 levels 24 h after intervention, whereas no significant alterations were found in MMP-2, -9, TIMP-1 and -2 levels. The question remains on how the increased activity of specific MMPs, in this case MMP-3 and -7, after endovascular recovery affects this process and whether they might be biomarkers of post-procedure outcomes or therapeutic targets [76].

Finally, midfoot amputation, performed simultaneously to distal revascularisation, potentially leads to major amputation, and significantly increases morbidity and mortality. Despite a successful reconstruction, the failure rate of minor amputations is up to 45%, and almost 30% of patients required a major amputation [77,78,79]. The healing progression is closely related to extracellular matrix synthesis and degradation and is mediated by MMPs. Specifically, it has been reported that MMP-2 and MMP-9 play a major role in this process regarding their affinity for basement membrane collagen type IV and laminin [80]. Sapienza P et al. analyzed plasma MMP-2 and MMP-9 levels in three groups of patients, those that underwent an infrapopliteal vein graft and midfoot amputation, others undergoing post-traumatic midfoot primary amputation without PAD, and in healthy controls with normal LDL-cholesterol levels and without atherosclerotic lesions (excluded by ultrasonography and ABI measurements). The postoperative high levels of MMP-2 and -9 were predictive of wound healing failure at three, six, and nine months in PAD patients. Furthermore, MMP-2, and -9 were even higher and more persistent in the subgroup of patients with occlusion of the vein graft at all tested time points. These results suggest that monitoring MMP-2 and MMP-9 might help in the identification of patients at risk of healing failure of midfoot amputation after distal revascularisation, and predict the fate of the vein graft [81].

MMPs have been involved in all stages of atherosclerosis, but also in matrix remodeling in restenosis processes post angioplasty. As such, their circulating levels have been evaluated as markers of PAD incidence, diagnosis and risk stratification by different authors. However, no clear consensus has been reached on which of the studied MMPs are the most promising for PAD assessment or whether this approach will benefit from the combination of several MMPs. Studies including different MMPs in larger cohorts with longer follow-up periods will need to be performed in order to clarify their utility in this regard.

## 4. Cardiac Damage Biomarkers in PAD

N-terminal pro-brain natriuretic peptide (NT-proBNP) and troponin (in particular high sensitivity troponin T, hsTnT), are the most accepted specific biomarkers of cardiac damage, which are released in conditions of cardiomyocyte stress and/or injury. While the mechanisms linking PAD and the cardiac release of these biomarkers are likely multifactorial, probably related to the high coexistence of PAD and CAD, and have not been fully elucidated, several studies have reported associations between these biomarkers and the evolution and prognosis of lower extremity PAD [10].

Recent data obtained in more than 12,000 subjects from the ARIC study showed that elevated NT-proBNP and hs-TnT levels were independently associated with incident symptomatic PAD (i.e., hospitalizations with PAD diagnosis or leg revascularization), especially in the cases of CLI [82]. Similarly, NT-proBNP was associated with incident symptomatic PAD in individuals from the cardiovascular cohort of the Malmo Diet and Cancer study [83], and it was also independently associated with PAD incidence in African-Americans and with the ABI in both African-Americans and non-Hispanic whites [84]. On the other hand, detectable hsTnT in the CAVASIC study (male patients with IC) was associated with an 84% higher probability of symptomatic PAD [85]. Moreover, in patients with chronic kidney disease from the CRIC study, hsTnT was independently associated with incident PAD over a mean follow-up of 7.4 years, and its addition to the Framingham risk score improved PAD discrimination [86]. Of note, within the PAD spectrum hsTnT levels were higher in patients with CLI than in those with IC [87].

Cardiac biomarkers have also shown prognostic value in PAD patients. Indeed, NT-proBNP has been reported as an independent predictor of mortality during a 5-year follow-up in symptomatic PAD patients from the LIPAD study [88,89]. It was also associated with higher rates of CV events, including CV mortality or hospitalization for myocardial infarction, stroke or coronary revascularization in male PAD patients [90]. In addition, the combination of NT-proBNP, CRP and average day pulse pressure added on top of relevant risk factors improved risk discrimination and net reclassification index in these patients [90]. Nevertheless, there are some conflicting data on the usefulness of this biomarker; whereas male patients with peripheral arterial occlusive disease who suffered a MACE during follow-up presented higher NT-proBNP levels at baseline, this association was not maintained in multivariable regression models [91]. In a relatively small study performed with 95 PAD patients, both NT-proBNP and hsTnT were associated with a higher risk of mortality, but after adjustment by age, gender, prior cerebral artery disease and diabetes mellitus only hsTnT remained statistically significant [92]. Interestingly, in receiver operating characteristics (ROC) analyses hsTnT, NT-proBNP and their combination were superior to carotid intima-media thickness and ABI for discriminating mortality risk [92]. Reinforcing the clinical usefulness of hsTnT in this context, in the CAVASIC study detectable hsTnT was associated with a higher risk of all-cause mortality and incident CV disease during a seven-year follow-up in adjusted models [85].

Finally, cardiac biomarkers might also provide some useful information on patient evolution after endovascular revascularization. In a large retrospective study with over 1,000 patients detectable hsTnT (>0.01 ng/mL) was associated with higher rates of mortality and amputation during a 1-year follow-up and this association was maintained after adjusting for potential confounding factors [93]. Similarly, after endovascular therapy for acute limb ischemia elevated hsTnT was associated with worse in-hospital outcomes (i.e., mortality or amputation) after adjusting for clinically relevant risk factors including history of CAD [94]. Moreover, myocardial injury after revascularization in CLI, defined by a plasma hsTnT ≥ 14ng/L and an increase of at least 30% from the baseline value was associated with a worse outcome, including MACE and mortality [95]. Interestingly, 85% of patients with hsTnT values reflecting myocardial injury did not have ischemic clinical symptoms or electrocardiography changes [95]. Regarding the usefulness of natriuretic peptides after endovascular revascularization, elevated BNP was an independent predictor of MACE during a 2 year follow-up, but it was not related to major adverse limb events (MALE) [96].

Therefore, cumulative evidence suggests that cardiac biomarkers may be clinically useful for the diagnosis of incident PAD as well as for providing prognostic information.

## 5. Extracellular Vesicles as Biomarkers in PAD 

Extracellular vesicles (EVs) are a heterogeneous population of small membranous particles that contain lipids, metabolites, proteins and nucleic acids from the cell of origin [97]. Their size and molecular content is determined by the type of biogenesis (i.e.,: multivesicular body exocytosis, plasma membrane budding or apoptosis) and the particular pathophysiological conditions at the time of their packaging and subsequent secretion into the extracellular space [98]. EVs in circulation contribute to the maintenance of vascular homeostasis and represent a promising component of liquid biopsy to identify novel biomarkers in CV diseases. In this review, following the last recommendations of the International Society for EVs [99], we will use the term EVs to refer to small and medium/large size vesicles also known as exosomes and microvesicles, respectively.

Circulating levels of EVs from different cellular origin are increased in response to CV risk factors (e.g., diabetes, hypertension, or hypercholesterolemia) and in patients with acute coronary syndromes, ischemic stroke or PAD [100]. Among them, platelet derived EVs (PEVs) constitute the major subtype of circulating EVs, possess high thrombogenic potential due to exposure of tissue factor and phosphatidylserine, and have been associated to atherosclerosis development and thrombosis [101]. Elevated numbers of PEVs have been found in symptomatic PAD patients compared to healthy subjects [102] and correlated to disease severity [103]. Moreover, PEVs subpopulations exposing P-selectin or CD63 were increased in PAD patients compared to age- and sex-matched controls and reflected the degree of platelet activation in vitro [104]. Endothelial EVs (EndEVs) are released into the blood flow upon endothelial injury or activation and their content could help to unravel molecular mechanisms that lead to endothelial and microcirculatory dysfunction in PAD [105]. Increased levels of circulating EndEVs have been found in several CV diseases such as stroke or CAD [106,107] being associated with endothelial dysfunction [108,109] and plaque instability [110,111]. Circulating EndEVs (CD144^+^) were found to be significantly upregulated in PAD patients, particularly those bearing the monomeric CRP isoform, suggesting their contribution to pro-inflammatory status of this disease [112]. Moreover, EVs from different cell origins, especially those of endothelial origin, expressing the pro-angiogenic Sonic hedgehog morphogen correlated with the number of collateral vessels in ischemic thighs of PAD patients suggesting their possible role in neovascularization [113]. In this regard, the number of EndEVs from skeletal muscles increased 2 days after femoral artery ligation in mice, and in vitro induced a more potent bone marrow–mononuclear cell differentiation towards an endothelial phenotype when compared to EVs isolated from control muscles. As such, in vivo, the co-injection of EVs from ischemic muscles and bone marrow–mononuclear cells potentiated the proangiogenic effect of the latter [114]. Similarly, another study found upregulated expression of several microRNAs (e.g., miR-21, miR-92a and miR-126) in circulating small EVs from PAD patients and showed their capacity to modulate migration of VSMCs and ECs in vitro [115].

Advances in high-throughput technologies have contributed to depict the heterogenous content of EVs and identify novel biomarkers and therapeutic targets in CV diseases [116], however, there is still scarce EVs-related -OMICs data regarding PAD pathophysiology. Recently, by the transcriptomic study of circulating medium/large size EVs we could identify 15 protein-coding genes differentially expressed between age- and sex-matched PAD patients and healthy controls [14]. Circulating EVs from CLI subjects were enriched in pro-inflammatory genes (e.g., *Lcn2* and *S100a9*) and transcripts related to signalling pathways of platelet biology, iron homeostasis and immune response. Moreover, serum levels of calprotectin (S100A8/A9 heterodimer) were elevated in PAD and associated with an increased risk of amputation and CV mortality during the follow-up. Overall, our results suggest that the application of high-throughput technologies to EVs might be helpful to identify new molecular targets for PAD diagnosis, outcome assessment, and treatment.

Although EVs have proven a remarkable potential for the identification of new biomarkers in PAD, their study still represents a technical challenge due to their small size and heterogenicity. Moreover, biological and technical factors such as medication, co-morbidities (e.g.,: aging, tobacco smoking) or EVs isolation method can influence both the number and the content of EVs [100,117]. For instance, cilostazol induced a reduction in the number of PEVs in PAD patients [118], while atorvastatin does not modify PEVs total numbers, but specific PEVs subpopulations; those exposing P-selectin, tissue factor and glycoprotein-IIIa compared to placebo-controls [119]. Interestingly, atorvastatin displayed the opposite effect on EndEVs inducing their increase in circulation [120]. These studies demonstrate that pharmacological treatments can alter both, the number and the cargo of EVs, and might consequently modify their functional role, highlighting the importance of considering factors that can potentially influence EVs bio-dynamics.

Circulating EVs represent a potential alternative for PAD evaluation. Likewise, changes in their absolute numbers, or in the numbers of specific EVs subpopulations have been associated to PAD stages, and the study of their content, reflecting the molecular changes induced by the proatherogenic/inflammatory stimuli, may be helpful for the identification of new diagnosis, prognosis and therapeutic targets. A major drawback for EVs application into clinical practice however, is the technical challenges related to their nanometric size and scarce biological cargo, that will be overcome by current and future technological advances.

## 6. Role of microRNAs in PAD 

MicroRNAs are small noncoding regulatory RNAs involved in the posttranscriptional modulation of gene expression. Increasing evidence suggest their involvement in the onset and progression of CV diseases, emerging as promising non-invasive biomarkers and therapeutic targets for several CV disorders [15] including PAD (Table 2).

Stather PW et al. performed the first whole miRNA transcriptomic analysis in blood of PAD subjects and validated 12 differentially expressed transcripts in two independent sets of PAD patients and healthy controls [121] (Table 2). Three of them, miR-16, -15b, and -363, exhibited high diagnostic value when assessed by ROC curve analysis. Recently, high-throughput sequencing of miRNAs in peripheral blood mononuclear cells of patients with lower extremity PAD revealed 29 differentially expressed miRNAs predicted to target protein-coding genes involved in pathologies of atherosclerotic aetiology [122]. Those 29 miRNAs presented good performance for PAD diagnosis by ROC curves and could effectively classify PAD patients and healthy subjects using unsupervised clustering methods [122]. Besides their potential as diagnostic biomarkers, miRNAs have also been associated with PAD severity. For example, circulating miR-210 and miR-124 appeared upregulated in PAD, and inversely correlated with claudication distance and ABI respectively [123,125]. Likewise, serum miR-27b and miR-130a were also increased in atherosclerosis obliterans patients and positively correlated with Fontaine stages [124].

miRNAs have been also determined to predict worse outcome and restenosis after surgery. For instance, miR-142 was increased in plasma (1.6-fold) and femoral plaques (3.4-fold) of PAD patients subjected to femoral bypass surgery suffering coronary artery stent implantation, any other heart or vascular surgical procedures, or toe or leg amputations within 1 year of follow-up compared to those without CV events. Moreover, plasma miR-142 independently predicted the occurrence of CV events during 1-year follow up after adjustment by age and sex [126]. In addition, according to Stojkovic S et al., miR-195 was found to independently predict the adverse atherothrombotic events and the need for target vessel revascularization after stent implantation during a two-year follow up. Moreover, miR-195 improved the predictive value of clinical risk factors including age, sex, active smoking, diabetes, hypertension and hyperlipidemia [127]. Two additional studies performed in PAD subjects also identified miR-143, -320a and -572 as potential prognostic biomarkers for in-stent restenosis [128,129].

miRNAs regulate multifactorial biological processes involved in the pathogenesis of PAD and its progression to CLI, including angiogenesis, arteriogenesis, inflammation, oxidative stress, and hypoxia [130]. Angiogenesis, a key cellular mechanism for tissue reperfusion, is tightly regulated by miRNAs in response to ischemic injury. For instance, miR-26b enhanced in-vivo angiogenesis in a murine microvasculature growth model, whereas it reduced muscle fiber necrosis after femoral artery ligation [131]. miR-126 is enriched in ECs and EVs from EPCs [132,133], and its inhibition impaired angiogenesis in the gastrocnemius muscle after hindlimb ischemia in mice [134]. Conversely, miR-16 has been shown to directly target the proangiogenic molecules vascular endothelial growth factor (VEGF), VEGF receptor-2, and fibroblast growth factor receptor (FGFR)-1, and impaired angiogenesis both in-vitro and in-vivo when overexpressed [135]. Furthermore, all these three miRNAs, miR-16, -26b and -126, were found downregulated (3- to 4-fold) in peripheral blood of PAD patients compared to healthy subjects, reinforcing their biological relevance in PAD pathophysiology [121]. In contrast, miR-210 has been found upregulated in serum of PAD patients [123]. Similarly, miR-210 was elevated in murine and gastrocnemius muscles after hindlimb ischemia, and its inhibition, accentuated skeletal muscle damage due to increased mitochondrial oxidative stress [136]. Arteriogenesis is also regulated by miRNAs. For instance, inhibition of four miRNAs (miR-329, -487b, -494, and -495) by gene silencing oligonucleotides was found to individually increase perfusion and collateral artery size and density in the adductor muscles of mice subjected to femoral artery ligation [137]. Moreover, intramuscular injection of anti-miR-146a after limb ischemia in mice resulted in increased blood flow recovery and collateralization in the hypoxic thighs [138]. Macrophages, major immune cells within the skeletal muscle, orchestrate the inflammatory response upon muscle ischemia and their regulation by miRNAs is fundamental for effective muscle regeneration [139]. Intramuscular injection of miR-27b mimic reduced infiltrating macrophage content and enhanced angiogenesis post-femoral artery ligation in mice [140]. miR-93 was also found to stimulate M2-like macrophage polarization and was associated with increased neovascularization and perfusion in the ischemic muscle of the miR-106b-93-25 cluster knock-out mice [141].

The studies summarized above suggest the possible use of miRNAs as circulating biomarkers for the diagnosis and prognosis of PAD. Moreover, they provide experimental insight into the molecular mechanism underlying arterial diseases, as the basis for future therapeutic targets. Despite these promising results, additional studies are still required to verify the clinical relevance of this findings. Moreover, the technical requirements of miRNA determination in blood, including RNA isolation, several PCR steps and the need of qualified personnel, might delay their application as routine laboratory test.

## 7. Machine Learning and PAD

Machine learning (ML) refers to computational methods based on statistical techniques and algorithms, that can model large datasets and detect useful patterns. In medicine, those prediction models can guide clinicians into the identification of subjects that might benefit from specific pharmacological or surgical interventions or aid estimating outcomes [16]. The application of ML methods to PAD might provide a great opportunity to improve patient classification and treatment, although currently research in this area is still scarce. Baloch ZQ et al. applied ML methods to explore the relationship between PAD degree, and functional limitation and symptoms severity. They found a nonlinear relationship between symptoms and effort tolerance amongst patients with and without PAD, that with a simple linear model would have been overlooked or considered unimportant. As such, ML models might contribute to identify asymptomatic PAD patients with great functional limitations, that otherwise would be lost to PAD diagnosis by regular tests [142]. Other authors described that the combination of proteomic data and clinical information rendered algorithms able to predict angiographically significant PAD [143]. Regarding CV risk stratification, Ross EG et al. reported that state of the art ML algorithms outperformed stepwise logistic regression models for the identification of PAD and the prognostication of mortality risk in this population [144]. More recently, they have demonstrated that the application of ML to electronic health records can generate learning-based models that accurately identify PAD patients at risk of future major adverse cardiac and cerebrovascular events [145]. In addition, ML algorithms might be useful to predict not only PAD medical burden, but also its associated financial cost. Likewise, Berger JS et al. applied ML methods to a retrospective CLI cohort and identified baseline predictors of subsequent one-year all-cause hospitalizations and total annual healthcare cost in this high risk patient subgroup [146]. PAD, a multifactorial pathology with complex interactions, might considerably benefit from methods able to integrate and analyze large datasets, including multiple biomarkers, providing prediction models leading to a better understanding of PAD pathophysiology and improving its diagnosis and risk assessment.

## 8. Conclusions

Despite its high prevalence, the lack of awareness in PAD clinical manifestations and the limited tools for its early diagnosis, progression and prognosis evaluation in a personalized manner, has resulted in suboptimal therapeutic interventions in all its stages. Non-invasive circulating biomarkers could be of value in this setting and several candidates have been evaluated as useful for the diagnosis and risk stratification of PAD. In addition, these studies have also provided insights into the pathophysiological mechanisms involved in PAD development and evolution. Of note, some of these biomarkers have been evaluated as possible targets for pharmacological interventions. However, more conclusive evidences into the causal relationship between the studied proteins and the growth and progress of lower limb atherosclerosis will be only obtained after clinical studies involving multicentric collaborations, larger cohorts and longer follow-up periods are completed. The analysis of circulating EVs might offer a new alternative for the discovery of novel biomarkers and therapeutic targets in PAD, while non-coding RNAs might provide useful information on the regulatory pathways governing atherosclerosis initiation and progression, as well as on ischemia-induced muscle damage. It is worth considering that PAD is a complex multifactorial pathology involving diverse molecular pathways. Consequently, the evaluation of a single biomarker might not reflect those complex interactions, being a multimarker approach more suitable for this purpose. In this line, machine learning methods might be useful to obtain more accurate prediction algorithms by combining numerous biomarkers and clinical and functional parameters. This will lead to earlier diagnosis of PAD, more accurate CV risk stratification, and more personalized pharmacological or surgical treatments.

## Figures and Tables

**Table 1:** Inflammatory biomarkers in lower limb PAD diagnosis and prognosis.

Assessed Biomarkers	Type of Biomarker	Studied Groups (n)	Outcome	Refs.
*CRP, D-dimer, fibrinogen, NT-proBNP and cTnT*	Prognosis	Systematic review and meta-analysis with 47 studies, 1990–2015. PAD patients (21,473). Minimum follow up 1 year.	Increased CRP (RR: 3.49, 95% CI: 2.35–5.19), D-dimer (RR: 2.22, 1.24–3.98), fibrinogen (RR: 2.08, 95% CI: 1.46–2.97), NT-proBNP (RR: 4.50, 95% CI: 2.98–6.81) and cTnT (RR: 3.33, 95% CI: 2.70–4.10) predicted risk of mortality in PAD patients. Association of CRP with MACE (RR: 1.86, 95% CI: 1.48–2.33).	[10]
*CRP*	Prognosis	Systematic review and meta-analysis with 16 studies, 2002–2017. Participants (5041). Minimum follow up 1 year.	Higher CRP levels predict MACE in PAD patients (HR: 1.38, 95% CI: 1.16–1.63, per unit increase in log_e_CRP).	[11]
*CRP*	Diagnosis Prognosis	PAD patients (317) and healthy controls (100). Mean follow up 3.6 years.	Increased CRP levels in PAD patients. Predictor of amputation (SHR: 1.76, 95% CI: 1.48–2.09) and MACE (amputation and CV mortality) (SHR: 1.53, 95% CI: 1.35–1.75).	[14]
*CRP*	Diagnosis	Prospective cohort (14916); symptomatic PAD (140) and healthy controls (140). Mean follow up 9 years.	Associated to incident PAD (RR: 2.8, 95% CI: 1.3–5.9).	[19]
*CRP*	Diagnosis	ARIC Study 1996–1998. Participants (9851), cases of PAD (316). Median follow up 17.4 years.	Associated to incident PAD and CLI (HR per 1 SD increase: 1.34, 95% CI: 1.18–1.52 and 1.34, 95% CI: 1.09–1.65, respectively).	[20]
*CRP, IL-6 & TNF-α*	Diagnosis	PAD patients (55) and healthy controls (34).	Increased CRP, IL-6 and TNF-α levels in PAD patients. IL-6 associated to PAD severity (ABI ≤ 0.90).	[21]
*CRP, IL-6, TNF-α & ICAM-1*	Diagnosis	PAD patients with intermittent claudication (75) and healthy subject (43).	Increased CRP, IL-6, TNF-α and ICAM-1 levels in PAD patients and inversely associated with maximal walking distance.	[22]
*CRP, IL-6, TNF-α & VCAM-1*	Diagnosis Prognosis	PAD patients (60) and healthy controls (50). Mean follow up of 2.24 years.	Increased CRP, IL-6 and TNF-α levels in PAD patients. CRP, IL-6, TNF-α and ICAM-1 associated with ABI. PAD patients with CRP > 1 mg/L had 4-fold higher risk of ischemic event or death.	[23]
*CRP, IL-6, ICAM-1 & D-dimer*	Diagnosis	PAD patients (62) and healthy controls (18).	Increased CRP, IL-6, ICAM-1 and D-dimer levels in PAD patients.	[24]
*CRP, IL-6, TNF-α, ICAM-1 & fibrinogen*	Diagnosis Prognosis	Framingham Offspring Study 1998–2001. Participants (2800), ABI < 0.9 (111).	CRP, IL-6, TNF-α and fibrinogen inversely associated to ABI. IL-6 related to ABI (OR: 1.21, 95% CI: 1.06–1.38) and intermittent claudication or lower extremity revascularization (OR: 1.36, 95% CI: 1.06–1.74).	[26]
*CRP, IL-6, ICAM-1 & VCAM-1*	Diagnosis	Edinburgh Artery Study 1988. Participants (2800). Follow up 5 and 12 years.	CRP, IL-6, ICAM-1 and VCAM-1 associated to PAD severity. IL-6 predicted ABI at 5 and 12 years.	[27]
*CRP*	Prognosis	PAD patients with (29) or without (38) adverse CV events. Follow up 5 years.	CRP levels were higher in PAD subjects with adverse CV events.	[28]
*CRP*	Prognosis	PAD patients (397). Average follow up 6.6 years.	CRP predicts total mortality at 2-years follow-up (HR = 1.56 per SD).	[29]
*CRP & D-dimer*	Prognosis	PAD patients (377). Follow up 4 years.	CRP and D-dimer predicts all-cause mortality within 1 and 2 years of follow-up (HR: 1.15, 95% CI: 1.06–1.24 and 1.14, 95% CI: 1.02–1.27, respectively).	[30]
*CRP, D-dimer & fibrinogen*	Diagnosis	PAD patients (45) and healthy controls (44).	CRP, D-dimer and fibrinogen were higher in PAD and associated to ABI.	[31]
*CRP*	Diagnosis	PAD patients (463). Mean follow up 6.1 years.	Higher CRP levels in patients with CLI compared to IC.	[25]
*CRP*	Prognosis	PAD patients (68). Follow up 6 months.	Pre- and post-operative (24 h) IL-6 levels and post-operative (24 h) CRP levels associated with six-month in-stent restenosis (OR: 1.11, 95% CI: 1.00–1.23, 1.04, 95% CI: 1.02–1.06 and 1.15, 95% CI: 1.04–1.26, respectively).	[32]
*IL-6, TNF-α, ICAM-1 & VCAM-1*	Diagnosis	PAD patients (20) and healthy controls (20).	Circulating IL-6, TNF-α, ICAM-1 and VCAM-1 levels were higher in PAD patients.	[33]
*IL-6, TNF-α, ICAM-1 & VCAM-1*	Diagnosis	PAD patients (80) and healthy controls (72).	All inflammatory and adhesion markers were higher in PAD patients.	[34]
*CRP, IL-6, ICAM-1, VCAM-1 and D-dimer*	Diagnosis	PAD patients (423).	CRP, IL-6, ICAM-1, VCAM-1 and D-dimer related to impaired lower limb functionality.	[35]
*IL-6*	Diagnosis	PAD patients (38). 1 year follow up.	Higher IL-6 levels were related to impaired walking distance.	[36]
*VCAM-1*	Diagnosis	PAD patients (51) and healthy controls (75).	VCAM-1 is increased in PAD patients.	[37]
*ICAM-1, VCAM-1 & D-dimer*	Diagnosis	PAD patients (60) and healthy controls (20).	ICAM-1, VCAM-1 and D-dimer increased in CLI patients.	[38]
*Fibrinogen*	Prognosis	FRENA registry. PAD patients (1363). Mean follow up 18 months.	High fibrinogen associated with ischemic events (HR: 1.61, 95% CI: 1.11–2.32) or major bleeding (HR: 3.42, 95% CI: 1.22–9.61).	[39]
*Fibrinogen*	Prognosis	LEADER trial 1992-2001. PAD patients (785). Follow up 3 years.	Fibrinogen predictor of death at 6 months (OR: 1.65, 95% CI: 0.96–2.73) and 3 years (OR: 1.44, 95% CI: 1.02–1.94).	[40]
*Fibrinogen*	Prognosis	PAD patients (486). Median follow up 7 years.	Fibrinogen levels predict risk of all-cause mortality (HR: 1.90, 95% CI: 1.11–3.41 for fibrinogen >12.2μmol/L) and CV death (HR: 2.68, 95% CI: 1.39–5.16 for fibrinogen >12.2 μmol/L).	[41]
*D-dimer*	Prognosis	BRAVO study 2009. PAD patients (595). Follow up 3 years.	D-dimer levels were increased in PAD patients 2 months before an ischemic heart event.	[42]
*NLR*	Diagnosis	PAD patients (733). Median follow-up 10.4 months.	Elevated NLR associated with severe PAD (OR: 1.07, 95% CI: 1.00–1.15).	[43]
*NLR*	Diagnosis	PAD patients (300).	NLR inversely associated with ABI.	[44]
*NLR*	Diagnosis	PAD patients (153) and controls (128).	NLR correlated to PAD severity.	[45]
*NLR*	Prognosis	CLI patients (172). Mean follow up 34.7 months.	NLR predicted amputation risk (HR: 1.14, 95% CI: 1.08–1.19).	[46]
*NLR*	Prognosis	PAD patients (593). Median follow-up 20 months.	High NLR (>3.0) was an independent predictor of long-term cardiovascular mortality (HR: 2.04, 95% CI: 1.26–3.30).	[47]
*NLR*	Prognosis	PAD patients (95). Follow up 2 years.	Postoperative high NLR (≥2.75) predicts target vessel revascularization (HR: 3.1, 95% CI: 1.3–7.7) in PAD subjects after angioplasty with stent implantation.	[48]
*NLR*	Prognosis	CLI patients (561). Median follow up 31 months.	Preoperative high NLR (>5) correlated with 5-year amputation-free survival (HR: 2.32, 95% CI 1.73–3.12) in PAD patients subjected to infrainguinal revascularization.	[49]
*NLR*	Prognosis	PAD patients (1228). Minimum follow up 1 year.	Preoperative NLR associated with MALE (HR: 1.09, 95% CI: 1.07–1.11) and 10-year mortality (HR: 1.09, 95% CI: 1.07–1.12) after revascularization (stenting/bypass graft).	[50]
*NLR*	Prognosis	PAD patients (83). Follow-up period 12 months.	PAD patients with high NLR (≥5.25) had increased risk of death (HR: 1.97, 95% CI: 1.08–3.62) compared with low NLR subjects (<5.25).	[51]

CRP, C reactive protein; IL-6, interleukin-6; TNF-α, tumor necrosis factor α; ICAM-1, intercellular adhesion molecule 1; VCAM-1, vascular cell adhesion molecule 1; NLR, neutrophil-to-lymphocyte ratio; ABI, Ankle brachial index; MACE, major adverse cardiovascular events; MALE, major adverse limb events; PAD, peripheral arterial disease; IC, intermittent claudication; CLI, Critic limb ischemia; HR, Hazard ratio; SHR, Sub-Hazard ratio; RR, Relative risk; SD, standard deviation.

**Table 2:** Circulating miRNAs as biomarkers in peripheral arterial disease (PAD).

Studied Groups (n)	Type of Biomarker	Sample Type	Candidate miRNAs	Refs.
PAD (20) and healthy controls (20)	Diagnostic	Whole blood	Among 12 miRNAs; miR-15b (AUC = 0.92), -16 (AUC = 0.93) and -363 (AUC = 0.93) had highest diagnostic value.	[121]
PAD (40) and healthy controls (19)	Diagnostic	PBMCs	29 miRNAs showed independent associations with PAD (AUC > 0.8 for all).	[122]
PAD (27) and healthy controls (27)	Diagnostic	Serum	miR-130a, -27b and -210 were upregulated in PAD miR-210 was inversely correlated with claudication distance.	[123]
ASO (104) and healthy controls (105)	Diagnostic	Serum	mir-130a and -27b were increased in ASO and positively correlated with disease severity.	[124]
PAD (49) and healthy controls (47)	Diagnostic	Whole blood	miR-124 negatively correlated with ABI.	[125]
PAD patients with (12) and without (35) CVEs; 1 year follow up after surgery.	Prognostic	Plasma	miR-142 predicted post-femoral bypass surgery associated CVEs; (AUC = 0.861).	[126]
PAD patients with intermittent claudication (62); 2 years after surgery.	Prognostic	Serum	miR-195 independently predicted adverse ischemic events (HR per 1-SD of 0.40, 95% CI: 0.23-0.68) and target vessel revascularization (HR per 1-SD of 0.40, 95% CI: 0.22-0.75) after angioplasty with stent implantation.	[127]
PAD (146) and healthy controls (62); follow up period not specified.	Prognostic	Plasma	miR-320a (AUC = 0.766) and -572 (AUC = 0.690) predicted in-stent restenosis.	[128]
PAD patients with (74) and without (91) in-stent restenosis; follow up period not specified.	Prognostic	Serum	Serum miR-143 was lower in restenosis group and predicted in-stent restenosis; AUC = 0.866.	[129]

CVE, Cardiovascular events; PBMC, Peripheral blood mononuclear cells; ASO, Atherosclerosis obliterans; ABI, Ankle brachial index.

## Abbreviations

ABI	Ankle brachial index
CAD	Coronary artery disease
CLI	Chronic limb ischemia
CRP	C reactive protein
CV	Cardiovascular
EML	Elastin degradation products
EPC	Endothelial progenitor cell
EVs	Extracellular vesicles
EndEVs	Endothelial Extracellular vesicles
FGFR	Fibroblast growth factor receptor
hsTnT	High sensitivity troponin T
IC	Intermittent claudication
ICAM	Intercellular Adhesion Molecule
IL	Interleukin
MACE	Major adverse cardiovascular event
MALE	Major adverse limb event
MCP	Monocyte chemoattractant protein
mi(cro)RNA	Micro ribonucleic acid
ML	Machine learning
MMP	Matrix metalloproteinase
NGAL	Neutrophil Gelatinase-Associated Lipocalin
NLR	Neutrophil-to-lymphocyte ratio
NT-proBNP	N-terminal pro-brain natriuretic peptide
PAD	Peripheral arterial disease
PEVs	Platelet extracellular vesicles
ROC	Receiver operating characteristics
TNF(R)	Tumor necrosis factor (receptor)
TIMP	Tissue inhibitor of matrix matalloproteinases
VCAM	Vascular cell adhesion molecule
VEGF(R)	Vascular endothelial growth factor (receptor)
VSMC	Vascular smooth muscle cell
